# Post-discontinuation Survival in Patients With Advanced NSCLC Receiving Immune Checkpoint Inhibitors: A Pooled Analysis of Prospective Cohort Studies

**DOI:** 10.1016/j.jtocrr.2025.100847

**Published:** 2025-05-15

**Authors:** Yusuke Inoue, Yoshihiro Kitahara, Masato Karayama, Kazuhiro Asada, Koji Nishimoto, Shun Matsuura, Dai Hashimoto, Masato Fujii, Takashi Matsui, Nao Inami, Mikio Toyoshima, Hiroyuki Matsuda, Masaki Ikeda, Mitsuru Niwa, Yusuke Kaida, Masaki Sato, Yasuhiro Ito, Hideki Yasui, Yuzo Suzuki, Hironao Hozumi, Kazuki Furuhashi, Noriyuki Enomoto, Tomoyuki Fujisawa, Naoki Inui, Takafumi Suda

**Affiliations:** a Second Division, Department of Internal Medicine, Hamamatsu University School of Medicine, 1-20-1 Handayama, Chuo-ku, Hamamatsu, Japan; b Department of Chemotherapy, Hamamatsu University School of Medicine, 1-20-1 Handayama, Chuo-ku, Hamamatsu, Japan; c Department of Respiratory Medicine, Shizuoka General Hospital, 4-27-1 Kita-ando, Aoi-ku, Shizuoka, Japan; d Department of Respiratory Medicine, Iwata City Hospital, 512-3 Ohkubo, Iwata, Japan; e Department of Respiratory Medicine, Fujieda Municipal General Hospital, 4-1-11 Surugadai, Fujieda, Japan; f Department of Pulmonary Medicine, Seirei Hamamatsu General Hospital, 2-12-12 Sumiyoshi, Chuo-ku, Hamamatsu, Japan; g Department of Respiratory Medicine, Shizuoka City Shizuoka Hospital, 10-93 Otemachi, Aoi-ku, Shizuoka, Japan; h Department of Respiratory Medicine, Seirei Mikatahara General Hospital, 3453 Mikatahara, Chuo-ku, Hamamatsu, Japan; i Department of Respiratory Medicine, Shizuoka City Shimizu Hospital, 1231 Miyakami, Shimizu-ku, Shizuoka, Japan; j Department of Respiratory Medicine, Hamamatsu Rosai Hospital, 25 Shougen-cho, Chuo-ku, Hamamatsu, Japan; k Department of Respiratory Medicine, Japanese Red Cross Shizuoka Hospital, 8-2 Otemachi, Aoi-ku, Shizuoka, Japan; l Department of Respiratory Medicine, Shizuoka Saiseikai General Hospital, 1-1-1 Oshika Shizuoka, Japan; m Department of Respiratory Medicine, Hamamatsu Medical Center, 328 Tomitsuka, Chuo-ku, Hamamatsu, Japan; n Department of Respiratory Medicine, Ensyu Hospital, 1-1-1 Chuou, Chuo-ku, Hamamatsu, Japan; o Department of Respiratory Medicine, Japanese Red Cross Hamamatsu Hospital, 1088-1 Kobayashi, Hamana-ku, Hamamatsu, Japan; p Department of Respiratory Medicine, NHO Tenryu Hospital, National Hospital Organization, 4201-2 Oro, Hamana-ku, Hamamatsu, Japan; q Department of Clinical Pharmacology and Therapeutics, Hamamatsu University School of Medicine, 1-20-1 Handayama, Chuo-ku, Hamamatsu, Shizuoka, Japan

**Keywords:** Immune checkpoint inhibitors, non-small cell lung cancer, discontinuation, post-discontinuation survival, immune-related adverse event

## Abstract

**Introduction:**

The safety of discontinuing immune checkpoint inhibitors (ICIs) because of a durable response in patients with advanced NSCLC remains uncertain, and post-discontinuation survival outcomes based on the reason for cessation are not well defined.

**Methods:**

A pooled analysis was conducted using data from four prospective cohort studies involving 835 patients with advanced NSCLC who discontinued ICIs. Patients were categorized based on discontinuation reasons: durable response; immune-related adverse events (irAEs) (subcategorized by tumor response at discontinuation); non-irAE adverse events; disease progression; and other causes.

**Results:**

Disease progression was the most common reason for ICI discontinuation (*N* = 528 [63.2%]), followed by irAEs (*N* = 187 [22.4%]) and tumor response (*N* = 23 [2.8%]). Regarding response status at ICI discontinuation due to irAEs, complete/partial response (CR/PR) was the most frequent (*N* = 85), followed by stable disease/not evaluable (SD/NE, *N* = 69) and disease progression (*N* = 33). After a median post-discontinuation follow-up of 15.8 months (interquartile range, 6.9–23.2), patients who discontinued because of a response had excellent outcomes, with no deaths and only three progression-free survival events. While post-discontinuation overall survival was comparable between the irAE-CR/PR and irAE-SD/NE groups, ICI therapy ≥12 months was associated with improved post-ICI discontinuation survival in the irAE-CR/PR group.

**Conclusions:**

Discontinuation of ICIs because of a durable tumor response is rare in real-world settings but represents a feasible strategy for patients with advanced NSCLC. Patients in the irAE-CR/PR group had favorable post-ICI discontinuation survival if they received ICI therapy lasting ≥12 months.

## Introduction

Immune checkpoint inhibitors (ICIs) targeting programmed death-1 (PD-1), programmed death ligand 1 (PD-L1), and cytotoxic T lymphocyte antigen 4 (CTLA-4) have become one of the main therapeutic options for advanced non-small cell lung cancer (NSCLC). ICIs offer durable responses in a subset of patients who require long-term repeated administrations over years. Among the key pivotal phase 3 trials evaluating the efficacy of ICIs in advanced NSCLC, a series of KEYNOTE trials, such as KEYNOTE-010,^1^ KEYNOTE-024,^2^ KEYNOTE-042,^3^ KEYNOTE-189,^4^ and KEYNOTE-407,^5^ limited the duration of pembrolizumab treatment for up to 2 years or 35 cycles every 3 weeks. Similarly, the phase 3 CheckMate 9LA^6^ and CheckMate 227^7^ trials, in which efficacy and safety of nivolumab plus ipilimumab with or without two cycles of chemotherapy, respectively, were assessed in treatment-naïve patients with NSCLC, the treatment duration of ICI therapy was limited to 2 years of follow-up. In contrast, other landmark phase 3 trials that evaluated the efficacy and safety of nivolumab (CheckMate 017^8^ and CheckMate 057^9^), nivolumab with chemotherapy and bevacizumab (ONO-4538-52/TASUKI-52^10^), atezolizumab with or without chemotherapy (OAK,^11^ IMpower110,^12^ IMpower130,^13^ IMpower132,^14^ and IMpower150^15^), and durvalumab plus tremelimumab combined with chemotherapy (POSEIDON^16^) did not limit the treatment duration of ICIs.

Notably, the CheckMate 153 trial showed that patients with previously treated advanced NSCLC had improved outcomes with continued nivolumab treatment compared with patients that discontinued treatment after 1 year.^17^ However, retrospective studies reported no statistically significant survival advantage for the indefinite-duration strategy compared with the 2-year fixed-duration strategy.^18^^,^^19^ Moreover, a 5-year pooled analysis of the CheckMate 017/057 trials indicated that the proportion of nivolumab-treated patients with previously treated NSCLC who remained alive stabilized at approximately 3 years and plateaued thereafter.^20^ Thus, how long ICI treatment should be continued and what proportion of patients with historically incurable advanced NSCLC discontinues ICIs because of ongoing clinical benefit in the real-world setting remain unknown.

ICI treatment is complicated by a spectrum of organ-specific immune-mediated toxicities, referred to as immune-related adverse events (irAEs). Some severe irAEs can be fatal, and many irAEs lead to the cessation of ICI treatment. While numerous studies have shown associations between irAE incidence and benefit from ICI therapy,^21^ patient prognosis after ICI discontinuation due to irAEs has not been fully explored.

In this pooled analysis of four prospective cohort studies, we aimed to provide real-world data on the reasons for discontinuation of ICI therapy in patients with advanced NSCLC. Additionally, we evaluated whether ICIs can be safely discontinued and assessed post-discontinuation survival outcomes on the basis of the reasons for therapy cessation.

## Material and Methods

### Study design and data acquisition

This retrospective, multicenter study integrated the data from four prospective cohort studies conducted in Japan. The study designs and eligibility criteria for three cohorts (cohorts A–C) were described previously.^22^, ^23^, ^24^ Briefly, patients with pathologically confirmed advanced or recurrent NSCLC who were scheduled for treatment with PD-1 inhibitors (cohort A), nivolumab (cohort B), and atezolizumab (cohort C) were enrolled. Cohort D included prospectively enrolled patients with advanced or recurrent lung cancer or pleural mesothelioma who were planned to receive nivolumab, pembrolizumab, or atezolizumab, primarily to prospectively assess the incidence of ICI-induced pneumonitis. Patients in cohort A were treated with single-agent nivolumab or pembrolizumab at Hamamatsu University Hospital between June 2016 and December 2018; those in cohort B were treated with single-agent nivolumab at 14 institutions between July 2016 and December 2018; those in cohort C were treated with atezolizumab with or without chemotherapy at 14 institutions between January 2019 and May 2020; and those in the cohort D were treated with ICIs with or without chemotherapy at 15 institutions between May 2019 and July 2023. The outcome data were updated in September 2024 (cohort A) and September 2023 (cohorts B and C). The dataset cutoff for cohort D was September 2024. The studies were registered at the University Hospital Medical Information Network (UMIN) Clinical Trials Registry as UMIN000023462 (cohort A), UMIN000022505 (cohort B), UMIN000035616 (cohort C), and UMIN000039761 (cohort D).

### Classification of ICI discontinuation patterns

We classified the reasons for ICI discontinuation as follows: physicians’ decision after achievement of a durable response, which included treatment discontinuation because of the completion of fixed-duration treatment; irAEs; non-irAE AEs; disease progression; and other causes. Discontinuation due to irAEs was classified by the tumor response at the time of treatment discontinuation: irAE-complete response/partial response (CR/PR); irAE-stable disease/not evaluable (SD/NE); and irAE-progressive disease (PD). In all cohorts (A–D), there were no predefined criteria for treatment discontinuation; decisions were made at the discretion of the treating physicians.

### Assessment of tumor response and AEs

Tumor responses were evaluated by local investigators using Response Evaluation Criteria in Solid Tumors (RECIST) version 1.1. AEs were classified using the Common Terminology Criteria for Adverse Events (CTCAE) version 5.0. We defined irAEs by the immune-mediated mechanism of action without any alternative etiology.^24^

### Lung immune prognostic index (LIPI) score calculation

LIPI at baseline was calculated by the derived neutrophil to lymphocyte ratio [dNLR; neutrophils/(leukocytes minus neutrophils)] and lactate dehydrogenase (LDH) level. dNLR greater than 3 and LDH greater than the upper limit of normal were scored as one; the sum was calculated for each case (score 0, 1, or 2).^25^

### Assessment of PD-L1 tumor proportion score (TPS)

For cohort B, tumor PD-L1 expression was assessed using the E1L3N antibody (Cell Signaling Technology, Danvers, MA, USA) or the 22C3 pharmDX assay (Agilent, Santa Clara, CA, USA) before and after the approval of the 22C3 assay in Japan, respectively; TPS was calculated as described previously.^23^ For the other cohorts, PD-L1 TPS was assessed using the 22C3 assay.

### Statistical analysis

The distributions of clinical factors were summarized as frequency (%) or median (range or interquartile range [IQR]). The date of ICI discontinuation was defined as the date of the last dose of ICIs; the duration of ICI therapy was defined as the time between the dates of the first and last ICI doses. Categorical variables were analyzed using Fisher’s exact test. Kaplan–Meier estimates and log-rank statistics were used to estimate and compare post-ICI discontinuation progression-free survival (PFS) and overall survival (OS). The survival durations were defined as the time between the date of the last administration of ICIs and the date of progression or death from any cause for post-ICI discontinuation PFS and the date of death from any cause for post-ICI discontinuation OS. Censoring was undertaken at the date of last contact. Hazard ratios (HRs) were estimated with Cox proportional hazards models. To explore the potential impact of treatment duration on outcomes in patients who discontinued ICI therapy due to irAEs, additional analyses were performed using 6-month and 12-month cutoffs for ICI treatment duration. These cutoff points were selected post hoc and were not pre-specified in the original study design. *P* values in multiple comparisons were adjusted using Holm’s method. Statistical tests were two-sided; *P* < .05 indicated statistical significance. Analyses were conducted using GraphPad Prism version 8.4.3 (GraphPad Software, San Diego, CA, USA) and EZR statistical software^26^ version 1.65 (Saitama Medical Center, Jichi Medical University).

### Ethics statement

This study was approved by the institutional review board at Hamamatsu University School of Medicine (#23-102) and conducted in accordance with the International Council for Harmonisation Good Clinical Practice guidelines and the Declaration of Helsinki. This study followed the Strengthening the Reporting of Observational Studies in Epidemiology (STROBE) reporting guidelines. Patient approval and written informed consent were waived because this study was based on reviews of patient records. Patients’ records and information were anonymized and de-identified prior to analysis. Information about the study was posted online (https://hama-med.bvits.com/rinri/publish.aspx) to provide the patients or their legal representative an opportunity to learn about the study and refuse participation (opt-out) if desired.

## Results

### Patient characteristics

**Figure 1:**
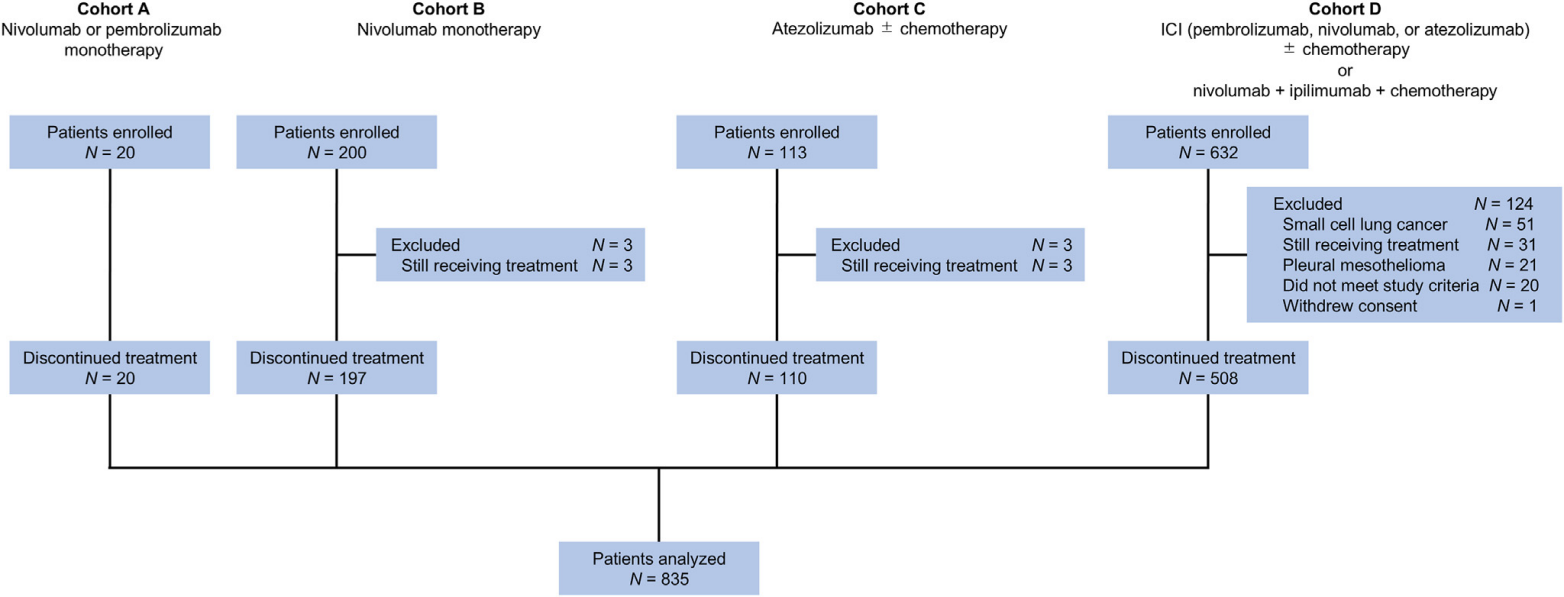
**Study schema.** ICI, immune checkpoint inhibitor.

Cohorts A–D initially comprised 20, 200, 113, and 632 patients, respectively. In cohorts B and C, three patients each were excluded because they were receiving ICIs at the dataset cutoff. In cohort D, 124 patients were excluded based on predefined criteria, including 31 who were still on ICI treatment at the cutoff date. Finally, 835 patients with NSCLC who discontinued ICI therapy were included in this study (Fig. 1). The baseline characteristics are summarized in Table 1. Most patients were male (76.2%), had an Eastern Cooperative Oncology Group (ECOG) performance status (PS) of 0 or 1 (92.4%), and had a history of smoking (79.6%). Adenocarcinoma was the most common histological subtype, accounting for 63.8% of cases. ICIs were administered as first-line, second-line, and third- or later-line therapy in 392 (46.9%), 228 (27.3%), and 215 (25.7%) patients, respectively. Single-agent ICIs were given for 476 patients (57.0%), while 342 (41.0%) received immunochemotherapy and 17 (2.0%) were treated with nivolumab and ipilimumab plus chemotherapy. Tumor PD-L1 expression was high (≥50%) in 228 patients (27.3%), low (1%–49%) in 251 (30.1%), and negative (<1%) in 280 (33.5%).

**Table 1:** Patient and tumor characteristics

Characteristic	Cohort A	Cohort B	Cohort C	Cohort D	Total
	*N* (%)	*N* (%)	*N* (%)	*N* (%)	*N* (%)
**No. of patients**					
	20	197	110	508	835
**Age, years**					
Median (range)	68 (40–82)	69 (43–83)	70 (36–84)	72 (36–90)	71 (36–90)
**Sex**					
Male	16 (80.0)	157 (79.7)	80 (72.7)	383 (75.4)	636 (76.2)
Female	4 (20.0)	40 (20.3)	30 (27.3)	125 (24.6)	199 (23.8)
**ECOG performance status**					
0	9 (45.0)	110 (55.8)	64 (58.2)	270 (53.1)	453 (54.3)
1	10 (50.0)	79 (40.1)	35 (31.8)	194 (38.2)	318 (38.1)
2	1 (5.0)	8 (4.1)	11 (10.0)	36 (7.1)	56 (6.7)
3	0	0	0	7 (1.4)	7 (0.8)
4	0	0	0	1 (0.2)	1 (0.1)
**Smoking status**					
Never	5 (25.0)	33 (16.8)	26 (23.6)	106 (20.9)	170 (20.4)
Current or former	15 (75.0)	164 (83.2)	84 (76.4)	402 (79.1)	665 (79.6)
**Histology**					
Adenocarcinoma	13 (65.0)	110 (55.8)	77 (70.0)	333 (65.6)	533 (63.8)
Squamous cell carcinoma	5 (25.0)	76 (38.6)	21 (19.1)	125 (24.6)	227 (27.2)
Other	2 (10.0)	11 (5.6)	12 (10.9)	50 (9.8)	75 (9.0)
**Stage at treatment**					
III	3 (15.0)	37 (18.8)	13 (11.8)	49 (9.6)	102 (12.2)
IV	17 (85.0)	138 (70.0)	88 (80.0)	392 (77.2)	635 (76.1)
Recurrence^a^	0	22 (11.2)	9 (8.2)	67 (13.2)	98 (11.7)
**ICI treatment line**^b^					
First	7 (35.0)	0	19 (17.3)	366 (72.0)	392 (46.9)
Second	8 (40.0)	100 (50.8)	48 (43.6)	72 (14.2)	228 (27.3)
Third or later	5 (25.0)	97 (49.2)	43 (39.1)	70 (13.8)	215 (25.7)
**Type of ICI**					
Nivolumab monotherapy	13 (65.0)	197 (100)	0	10 (2.0)	220 (26.3)
Atezolizumab monotherapy	0	0	84 (76.4)	71 (14.0)	155 (18.6)
Pembrolizumab monotherapy	7 (35.0)	0	0	94 (18.5)	101 (12.1)
PD-(L)1 inhibitors + chemotherapy ± bevacizumab	0	0	26 (23.6)	316 (62.2)	342 (41.0)
Nivolumab + ipilimumab + chemotherapy	0	0	0	17 (3.3)	17 (2.0)
**Previous treatment with PD-(L)1 inhibitors**					
Yes	0	0	10 (9.1)	13 (2.6)	23 (2.8)
No	20 (100)	197 (100)	100 (90.9)	495 (97.4)	812 (97.2)
***EGFR* mutation**					
Presence	1 (5.0)	17 (8.6)	25 (22.7)	86 (16.9)	129 (15.4)
Absence	19 (95.0)	138 (70.1)	71 (64.6)	352 (69.3)	580 (69.5)
Unknown	0	42 (21.3)	14 (12.7)	70 (13.8)	126 (15.1)
***ALK* rearrangement**					
Presence	0	1 (0.5)	0	7 (1.4)	8 (0.9)
Absence	18 (90.0)	141 (71.6)	85 (77.3)	407 (80.1)	651 (78.0)
Unknown	2 (10.0)	55 (27.9)	25 (22.7)	94 (18.5)	176 (21.1)
**PD-L1 status**					
≥50%	8 (40.0)	23 (11.7)	16 (14.5)	181 (35.6)	228 (27.3)
1%–49%	6 (30.0)	65 (33.0)	40 (36.4)	140 (27.6)	251 (30.1)
<1%	5 (25.0)	109 (55.3)	39 (35.5)	127 (25.0)	280 (33.5)
Unknown	1 (5.0)	0	15 (13.6)	60 (11.8)	76 (9.1)

*ALK*, anaplastic lymphoma kinase; ECOG, Eastern Cooperative Oncology Group; *EGFR*, epidermal growth factor receptor; ICI, immune checkpoint inhibitor; NOS, not otherwise specified; PD-1, programmed death-1; PD-L1, programmed death-ligand 1.

a Includes recurrence after surgery, chemoradiotherapy, and radiotherapy.

b Excludes (neo)adjuvant chemotherapy for surgical cases and chemoradiotherapy ± durvalumab for stage III disease from prior treatments.

### Treatment discontinuation due to tumor response

**Figure 2:**
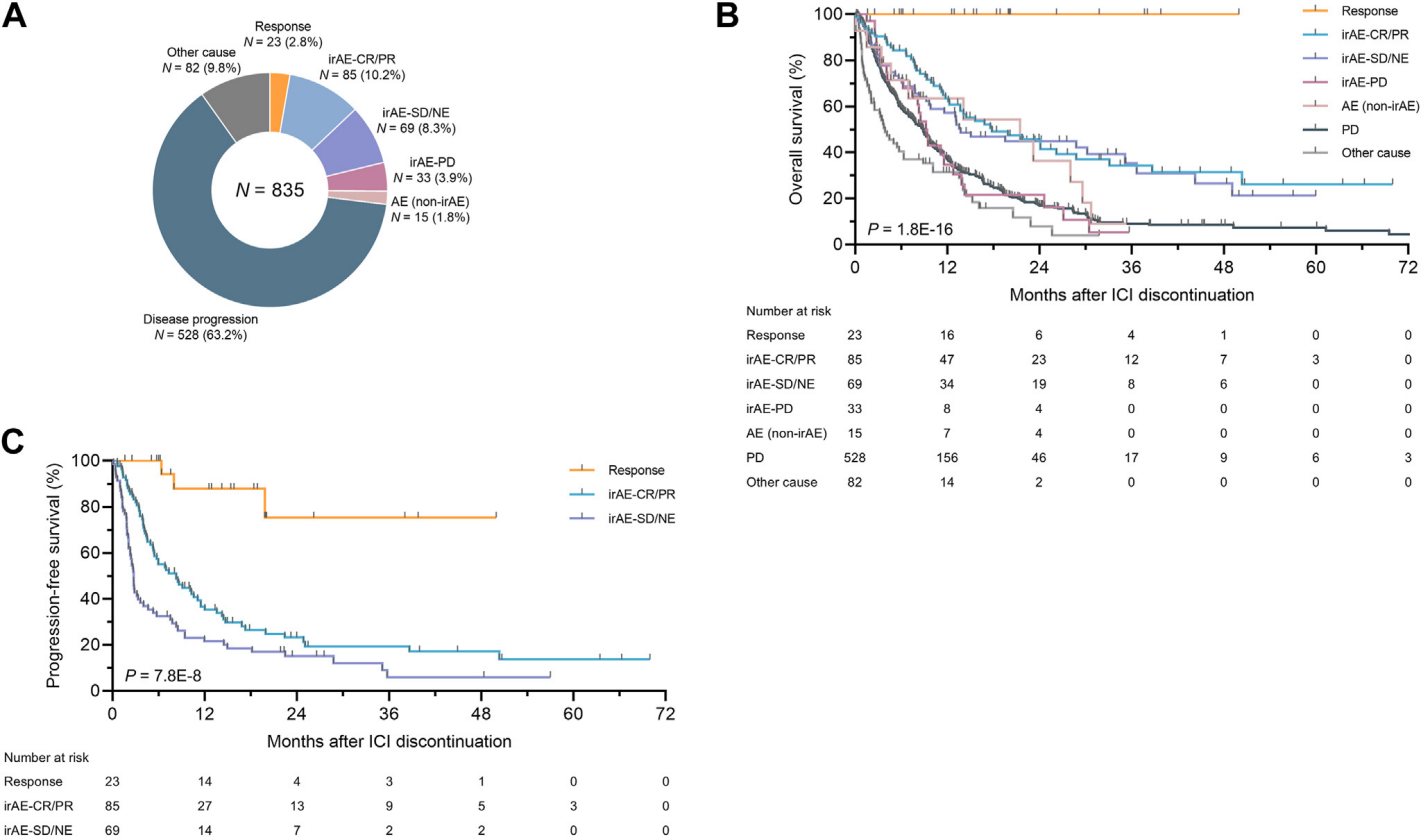
**Patterns of ICI discontinuation and post-ICI discontinuation survival outcomes on the basis of reasons for treatment discontinuation in patients with NSCLC. (a)** Pie chart representing the proportions of ICI discontinuation patterns in patients with NSCLC (*N* = 835). **(b and c)** Kaplan–Meier plots of post-ICI discontinuation overall survival **(b)** and progression-free survival **(c)** in patients categorized by reasons for ICI discontinuation. CR, complete response; ICI, immune checkpoint inhibitor; irAE, immune-related adverse event; NE, not evaluable; NSCLC, non-small cell lung cancer; PD, progressive disease; PR, partial response; SD, stable disease.

Disease progression was the most frequent cause of ICI discontinuation (*N* = 528 [63.2%]), followed by irAEs (*N* = 187 [22.4%]) and tumor response (*N* = 23 [2.8%]) (Fig. 2*A*). Other causes (*N* = 82 [9.8%]) included deterioration in PS without meeting the criteria for PD (*N* = 41), comorbidities (*N* = 11), death (*N* = 10), patient withdrawal (*N* = 10), and other reasons (*N* = 10). Among the 36 patients who received ICIs and remained progression-free at 2 years, ICIs were discontinued because of sustained responses in 14 patients (38.9%).

A summary of the 23 patients who discontinued ICIs because of tumor responses is presented in Table 2. Among the 23 patients, 21 (91.3%) were male, 22 (95.7%) had a smoking history, and 17 (73.9%) had adenocarcinoma histology. No tumors harbored *EGFR* mutations and *ALK* rearrangements. Tumor PD-L1 TPS was high (≥50%) in 14 patients (60.9%) and negative in three (13.0%). ICIs were given as monotherapy in 10 patients (43.5%), among whom 5 tumors (50.0%) showed high PD-L1 expression. Most patients received ICIs as first-line treatment (*N* = 18, 78.3%). The best response was PR in 12 patients (52.2%), followed by CR in 9 (39.1%), and SD in 2 (8.7%). At the time of ICI discontinuation, the response status was PR in 11 patients (47.8%), CR in 9 (39.1%), and SD in 3 (13.0%). The median duration of ICI therapy was 25.1 months (IQR, 23.7–32.3); ICIs were discontinued after more than 24 months of treatment in 15 patients (65.2%). irAEs including hypothyroidism (*N* = 2), adrenal insufficiency (*N* = 1), and pneumonitis (*N* = 1) were documented in four patients (17.4%).

**Table 2:** Patient and tumor characteristics of patients who discontinued ICI treatment due to response

Patient identification	Sex	Age (years)	Smoking status	Histology	Stage	PD-L1 TPS	ICI treatment line	ICI	ICI monotherapy	Best response	Response at ICI discontinuation	ICI duration (months)	irAE	Type of irAE
1	Female	71	Current	ADC	3	≥50%	1	Pemb	No	CR	CR	47.5	Yes	Adrenal insufficiency
2	Male	63	Former	SCC	Recurrent	<1%	1	Pemb	No	CR	CR	25.1	No	
3	Female	66	Never	ADC	4	≥50%	1	Atez	No	PR	PR	8.6	No	
4	Male	73	Former	SCC	Recurrent	1%–49%	1	Pemb	No	PR	PR	24.2	No	
5	Male	68	Former	ADC	4	NA	1	Pemb	No	CR	CR	11.3	No	
6	Male	75	Former	ADC	4	≥50%	1	Pemb	No	PR	SD	26.7	No	
7	Male	76	Former	SCC	4	≥50%	1	Pemb	No	CR	CR	26.8	Yes	Pneumonitis
8	Male	72	Former	ADC	Recurrent	≥50%	1	Pemb	Yes	PR	PR	24.2	No	
9	Male	71	Former	ADC	Recurrent	NA	2	Atez	Yes	PR	PR	6.9	No	
10	Male	72	Former	ADC	4	≥50%	1	Pemb	No	CR	CR	25.7	No	
11	Male	78	Current	ADC	4	≥50%	1	Pemb	Yes	SD	SD	23.7	No	
12	Male	69	Former	Large	4	≥50%	1	Pemb	No	CR	CR	38.1	No	
13	Male	64	Former	ADC	4	<1%	1	Pemb	No	PR	PR	34.8	No	
14	Male	70	Former	ADC	4	≥50%	1	Pemb	No	SD	SD	24.5	No	
15	Male	61	Current	ADC	4	≥50%	1	Pemb	No	PR	PR	29.8	No	
16	Male	80	Former	ADC	4	≥50%	1	Pemb	Yes	PR	PR	19.4	No	
17	Male	81	Former	ADC	4	≥50%	1	Pemb	Yes	PR	PR	23.8	Yes	Hypothyroidism
18	Male	74	Former	NOS	4	≥50%	1	Pemb	Yes	CR	CR	23.7	No	
19	Male	63	Former	NOS	4	≥50%	1	Pemb	No	CR	CR	2.8	No	
20	Male	69	Current	ADC	4	NA	2	Niv	Yes	PR	PR	37.2	No	
21	Male	49	Current	ADC	4	NA	2	Niv	Yes	PR	PR	57.3	Yes	Hypothyroidism
22	Male	79	Former	ADC	4	NA	2	Atez	Yes	CR	CR	26.7	No	
23	Male	55	Former	ADC	4	<1%	3	Atez	Yes	PR	PR	35.6	No	

ADC, adenocarcinoma; Atez, atezolizumab; CR, complete response; ICI, immune checkpoint inhibitor; irAE, immune-related adverse event; large, large cell carcinoma; NA, not available; Niv, nivolumab; NOS, not otherwise specified; PD-L1, programmed death-ligand 1; Pemb, pembrolizumab; PR, partial response; SCC, squamous cell carcinoma; SD, stable disease;; TPS, tumor proportion score.

### Treatment discontinuation due to irAEs

A total of 344 irAEs occurred in 290 patients (34.7%), and ICI therapy was discontinued due to irAEs in 187 patients (22.4%). The most common irAEs that resulted in ICI discontinuation were pneumonitis (*N* = 100 [53.5%]), colitis (*N* = 20 [10.7%]), and rash (*N* = 12 [6.4%]) (Table 3). Regarding the response status at ICI discontinuation due to irAEs, CR/PR was the most frequent (*N* = 85 [45.5%]), followed by SD/NE (*N* = 69 [36.9%]) and PD (*N* = 33 [17.6%]) (Fig. 2*A*, Table 3).

**Table 3:** Summary of irAEs leading to ICI discontinuation on the basis of response status at the time of ICI discontinuation

irAE	Total (*N* = 187)		irAE-CR/PR (*N* = 85)		irAE-SD/NE (*N* = 69)		irAE-PD (*N* = 33)	
	Any grade	Grade ≥3	Any grade	Grade ≥3	Any grade	Grade ≥3	Any grade	Grade ≥3
	*N* (%)	*N* (%)	*N* (%)	*N* (%)	*N* (%)	*N* (%)	*N* (%)	*N* (%)
**Respiratory**	100 (53.5)	38 (20.3)	43 (50.6)	17 (20.0)	43 (62.3)	15 (21.7)	14 (42.4)	6 (18.2)
Pneumonitis	100 (53.5)	38 (20.3)	43 (50.6)	17 (20.0)	43 (62.3)	15 (21.7)	14 (42.4)	6 (18.2)
**Gastrointestinal**	33 (17.6)	11 (5.9)	15 (17.6)	5 (5.9)	10 (14.5)	3 (4.3)	8 (24.2)	3 (9.1)
Colitis	20 (10.7)	4 (2.1)	9 (10.6)	2 (2.4)	8 (11.6)	2 (2.9)	3 (9.1)	0
Hepatitis	10 (5.3)^a^	5 (2.7)^a^	5 (5.9)	2 (2.4)	1 (1.4)	1 (1.4)	4 (12.1)^a^	2 (6.1)^a^
Sclerosing cholangitis	2 (1.1)	1 (0.5)	0	0	1 (1.4)	0	1 (3.0)	1 (3.0)
Duodenal ulcer	1 (0.5)	1 (0.5)	1 (1.2)	1 (1.2)	0	0	0	0
**Endocrine**	13 (7.0)	5 (2.7)	7 (8.2)	3 (3.5)	3 (4.3)	1 (1.4)	3 (9.1)	1 (3.0)
Adrenal insufficiency	7 (3.7)	3 (1.6)	4 (4.7)	2 (2.4)	0	0	3 (9.1)	1 (3.0)
Thyroiditis	2 (1.1)	0	1 (1.2)	0	1 (1.4)	0	0	0
Hypothyroidism	2 (1.1)	0	1 (1.2)	0	1 (1.4)	0	0	0
Type 1 diabetes	2 (1.1)	2 (1.1)	1 (1.2)	1 (1.2)	1 (1.4)	1 (1.4)	0	0
**Cutaneous**	13 (7.0)	8 (4.3)	5 (5.9)	2 (2.4)	4 (5.8)	4 (5.8)	4 (12.1)	2 (6.1)
Rash	12 (6.4)^b^	8 (4.3)	4 (4.7)	2 (2.4)	4 (5.8)	4 (5.8)	4 (12.1)^b^	2 (6.1)
Pruritus	1 (0.5)	0	1 (1.2)	0	0	0	0	0
**Musculoskeletal or rheumatic**	10 (5.3)	4 (2.1)	6	2 (2.4)	2 (2.9)	1 (1.4)	2 (6.1)	1 (3.0)
Arthritis	3 (1.6)	0	3 (3.5)	0	0	0	0	0
Myositis	3 (1.6)^a^	1 (0.5)^a^	1 (1.2)	0	0	0	2 (6.1)^a^	1 (3.0)^a^
Connective tissue disease	3 (1.6)	2 (1.1)	1 (1.2)	1 (1.2)	2 (2.9)	1 (1.4)	0	0
Myalgia	1 (0.5)	1 (0.5)	1 (1.2)	1 (1.2)	0	0	0	0
**Renal**	6 (3.2)	2 (1.1)	4 (4.7)	1 (1.2)	1 (1.4)	0	1 (3.0)	1 (3.0)
Nephritis	6 (3.2)	2 (1.1)	4 (4.7)	1 (1.2)	1 (1.4)	0	1 (3.0)	1 (3.0)
**Neurological**	6 (3.2)	6 (3.2)	1 (1.2)	1 (1.2)	1 (1.4)	1 (1.4)	4 (12.1)	4 (12.1)
Myasthenia gravis	4 (2.1)^a^^,^^b^	4 (2.1)^a^	1 (1.2)	1 (1.2)	0	0	3 (9.1)^a^^,^^b^	3 (9.1)^a^
Aseptic meningitis	1 (0.5)	1 (0.5)	0	0	1 (1.4)	1 (1.4)	0	0
Acute motor axonal neuropathy	1 (0.5)	1 (0.5)	0	0	0	0	1 (3.0)	1 (3.0)
**Hematologic**	2 (1.1)	2 (1.1)	1 (1.2)	1 (1.2)	1 (1.4)	1 (1.4)	0	0
Hemophagocytic lymphohistiocytosis	2 (1.1)^c^	2 (1.1)^c^	1 (1.2)^c^	1 (1.2)^c^	1 (1.4)	1 (1.4)	0	0
Autoimmune hemolytic anemia	1 (0.5)^c^	1 (0.5)^c^	1 (1.2)^c^	1 (1.2)^c^	0	0	0	0
**Other**	8 (4.3)	5 (2.7)	3 (3.5)	3 (3.5)	4 (5.8)	2 (2.9)	1 (3.0)	0
Fever	3 (1.6)^b^	0	0	0	2 (2.9)	0	1 (3.0)^b^	0
Mucositis	3 (1.6)	3 (1.6)	2 (2.4)	2 (2.4)	1 (1.4)	1 (1.4)	0	0
Cytokine release syndrome	1 (0.5)	1 (0.5)	1 (1.2)	1 (1.2)	0	0	0	0
T-cell lymphoma	1 (0.5)	1 (0.5)	0	0	1 (1.4)	1 (1.4)	0	0

CR, complete response; ICI, immune checkpoint inhibitor; irAE, immune-related adverse event; NE, not evaluable; PD, progressive disease; PR, partial response; SD, stable disease.

a Hepatitis, myositis, and myasthenia gravis developed in a patient.

b Rash, myasthenia gravis, and fever developed in a patient.

c Autoimmune hemolytic anemia, and hemophagocytic lymphohistiocytosis developed in a patient.

The median duration of ICI therapy was 3.9 months (IQR, 1.4–7.8) in the irAE-CR/PR group, with median durations of 3.7 months (IQR, 1.3–6.5), 2.1 months (IQR, 1.0–7.3), and 5.5 months (IQR, 3.5–7.9) in the first-line, second-line, and third- or later-line settings, respectively. In the irAE-SD/NE group, the median duration was 0.9 months (IQR, 0–3.2), with corresponding values of 1.6 months (IQR, 0–3.9), 0.9 months (IQR, 0.3–2.2), and 0.5 months (IQR, 0–2.1) by line of therapy. In the irAE-PD group, the overall median duration was 1.6 months (IQR, 0–8.9), with medians of 6.1 months (IQR, 1.7–9.6), 0.8 months (IQR, 0–3.5), and 0.5 months (IQR, 0–3.2) in the first-, second-, and third- or later-line settings, respectively.

### Clinical factors at baseline associated with ICI discontinuation patterns

**Supplementary Figure S1:**
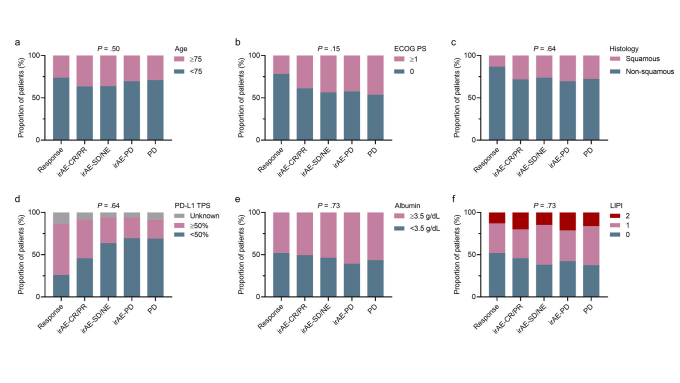
**Patient characteristics at baseline on the basis of reasons for ICI discontinuation**. **(a)** Proportion of patients ≥75 or <75 years old on the basis of ICI discontinuation patterns. **(b)** Proportion of patients with an ECOG PS of ≥1 or 0 on the basis of ICI discontinuation patterns. **(c)** Proportion of patients with squamous cell carcinoma or non-squamous histology on the basis of ICI discontinuation patterns. **(d)** Proportion of patients with PD-L1 TPS of ≥50%, <50%, or unknown on the basis of ICI discontinuation patterns. **(e)** Proportion of patients with a baseline serum albumin level of >3.5 g/dL or ≤3.5 g/dL on the basis of ICI discontinuation patterns. **(f)** Proportion of patients with an LIPI of 2, 1, or 0 on the basis of ICI discontinuation patterns. *P* value was calculated using Fisher’s exact test. CR, complete response; ECOG, Eastern Cooperative Oncology Group; ICI, immune checkpoint inhibitor; irAE, immune-related adverse event; LIPI, lung immune prognostic index; NE, not evaluable; PD, progressive disease; PR, partial response; PS, performance status; SD, stable disease.

We next assessed whether clinical factors at the time of ICI treatment initiation were associated with the major ICI discontinuation patterns such as response, irAE-CR/PR, irAE-SD/NE, irAE-PD, and PD. However, no significant associations were observed between ICI discontinuation patterns and patient age, ECOG PS, tumor histology, PD-L1 TPS, serum albumin levels, and LIPI (Supplementary Fig. S1).

### Post-ICI discontinuation survival

The median durations of follow-up after ICI discontinuation were 15.8 months (IQR, 6.9–23.2) for patients who discontinued ICIs because of a response, 13.7 months (IQR, 7.6–24.1) for irAE-CR/PR patients, 11.6 months (IQR, 4.6–26.6) for irAE-SD/NE patients, 8.3 months (IQR, 3.6–11.6) for irAE-PD patients, 7.5 months (IQR, 4.0–25.6) for AE patients, and 7.0 months (IQR, 3.0–13.0) for patients who discontinued ICIs because of PD. In patients who discontinued ICIs because of a response, no deaths (Fig. 2*B*) and three events of disease progression (Fig. 2*C*) were observed during the follow-up period. The durations of ICI therapy in the three patients whose tumors progressed after discontinuation were 6.9, 24.2, and 25.1 months. There were significant differences in post-ICI discontinuation OS by the discontinuation patterns (log-rank, *P* = 1.8E-16; Fig. 2*B*). However, there was no significant difference between the irAE-CR/PR (median, 17.8 months; 95% CI, 12.3–28.8) and irAE-SD/NE (median, 13.7 months; 95% CI, 9.3–35.1) groups (log-rank adjusted by Holm’s method, *P* = 1.00). Post-discontinuation OS outcomes were similarly unfavorable between the irAE-PD and PD groups (log-rank adjusted using the method of Holm, *P* = 1.00), and the median survival duration for the irAE-PD group (median, 9.4 months; 95% CI, 6.2–12.8) was significantly shorter than that of the irAE-CR/PR group (log-rank adjusted by Holm’s method, *P* = .012). When analyzing post-ICI discontinuation PFS, significant differences were observed among the response, irAE-CR/PR, and irAE-SD/NE groups (log-rank, *P* = 7.8E-8; Fig. 2*C*). Notably, the median post-ICI discontinuation PFS was significantly longer in the irAE-CR/PR group (8.3 months; 95% CI, 5.3–11.1) compared with the irAE-SD/NE group (2.8 months; 95% CI, 2.1–3.6; log-rank adjusted by Holm’s method, *P* = .0022).

### Post-ICI survival outcomes after discontinuing treatment because of irAEs without disease progression

**Figure 3:**
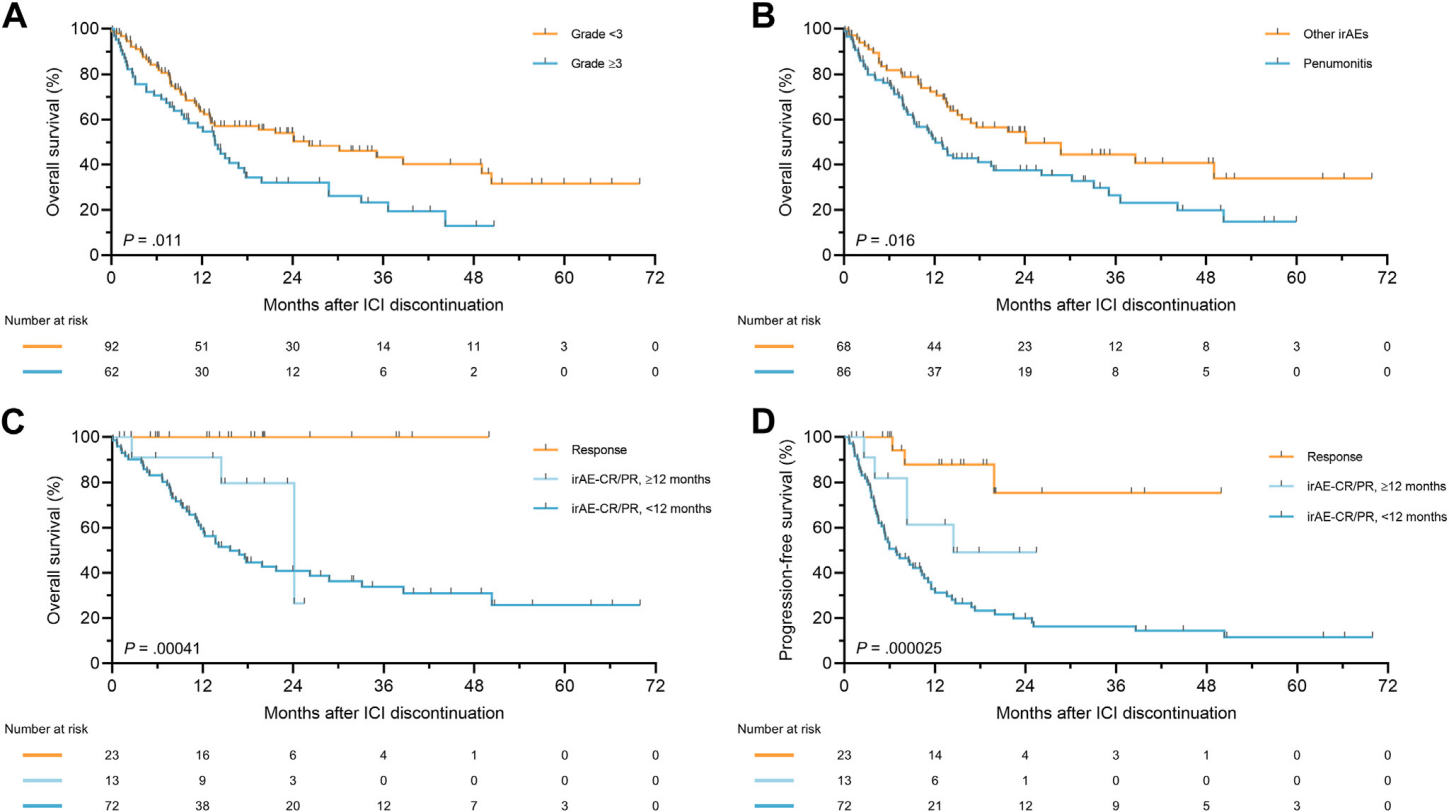
**Post-ICI discontinuation survival outcomes in patients with NSCLC who stopped ICI treatment because of irAEs. (a)** Kaplan–Meier plots of post-ICI discontinuation overall survival according to the severity of irAEs (grade ≥3 vs grade <3). **(b)** Kaplan–Meier plots of post-ICI discontinuation overall survival according to the type of irAEs (pneumonitis vs others). **(c and d)** Kaplan–Meier plots of post-ICI discontinuation overall survival **(c)** and progression-free survival **(d)** according to the duration of ICI treatment (≥12 months vs <12 months) in patients who stopped therapy because of irAEs with tumor response at the time of discontinuation. Survival curves in patients who stopped ICI treatment because of responses are also shown. CR, complete response; ICI, immune checkpoint inhibitor; irAEs, immune-related adverse events; NSCLC, non-small cell lung cancer; PR, partial response.

We next evaluated post-ICI survival outcomes in patients who discontinued ICIs because of irAEs without PD, categorized as the irAE-CR/PR and irAE-SD/NE groups. In the comparison of post-ICI discontinuation OS by the severity of irAEs leading to treatment cessation, patients experiencing grade <3 irAEs demonstrated significantly better outcomes than those with grade ≥3 irAEs (log-rank, *P* = .011; HR, 0.58; 95% CI, 0.38–0.89; Fig. 3*A*). Considering the potential adverse prognostic impact of pneumonitis among various irAEs,^21^ we also analyzed post-ICI OS by the type of irAE (pneumonitis vs. others). Patients who discontinued ICIs because of non-pneumonitis irAEs exhibited longer survival compared with those with pneumonitis (log-rank, *P* = .016; HR, 0.59; 95% CI, 0.39–0.91; Fig. 3*B*).

**Supplementary Figure S2:**
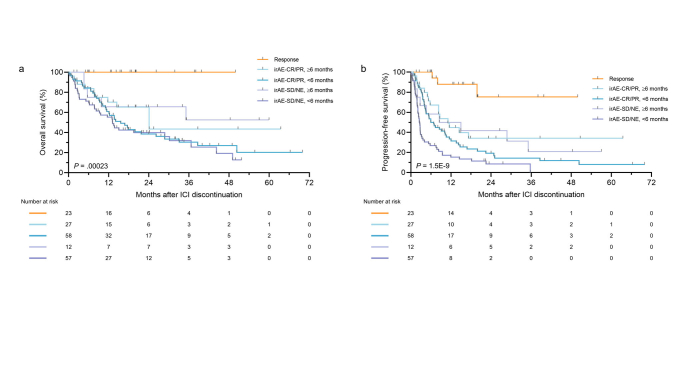
**Post-ICI discontinuation survival outcomes according to the duration of ICI treatment (≥6 months vs <6 months) in patients with NSCLC who stopped therapy because of irAEs without disease progression at the time of ICI discontinuation.** Kaplan–Meier plots of post-ICI discontinuation overall survival **(a)** and progression-free survival **(b)**. Survival curves in patients who stopped ICI treatment because of responses are also shown. ICI, immune checkpoint inhibitor; irAEs, immune-related adverse events. CR, complete response; NE, not evaluable; NSCLC, non-small cell lung cancer; PD, progressive disease; PR, partial response; SD, stable disease.

Next, we explored whether ICI therapy duration was associated with post-ICI survival in patients who discontinued ICI therapy because of irAE without disease progression. We first divided the patients at the 6-month cutoff. While survival was significantly different between the response group and other groups, post-ICI discontinuation OS (Supplementary Fig. S2*A*) and PFS (Supplementary Fig. S2*B*) did not significantly differ within the irAE-CR/PR group or within the irAE-SD/NE group when stratified by ICI treatment duration using this cutoff. When next divided patients at the 12-month cutoff of ICI therapy duration, we excluded irAE-SD/NE patients because of the small number of patients with irAE-SD/NE who received ICI therapy for ≥12 months (*N* = 4). Despite still being significantly shorter than post-ICI discontinuation OS of the response group (log-rank adjusted by Holm’s method, *P* = .0093; Fig. 3*C*), the median survival time of the irAE-CR/PR group with ICI therapy duration of ≥12 months was 24.1 months (95% CI, 14.5–not reached), compared with 15.6 months (95% CI, 11.2–28.8) in the group with ICI therapy duration <12 months. In terms of post-ICI discontinuation PFS, there was no significant difference between the response group and the irAE-CR/PR group with ≥12 months of ICI therapy (log-rank adjusted by Holm’s method, *P* = .13; Fig. 3*D*).

## Discussion

Given the exponential rise in ICI use and durable remissions observed in a subset of patients with NSCLC, determining the optimal treatment duration is an important question. In melanoma, where the CR rate is higher,^27^ stopping ICIs after achieving durable remissions is particularly relevant to mitigate both the physical and financial toxicities associated with years of treatment.^28^ In this study, we found that the rate of elective ICI discontinuing because of a response in real-world NSCLC cohorts was 2.8% (23 of 835 patients). While this represents a minority of patients overall, among those who remained progression-free at 2 years on ICI therapy, approximately 40% discontinued ICIs because of a response. This proportion was apparently higher than that (16%) reported in a retrospective cohort study conducted in the USA.^18^

Consistent with the upper limits for the duration of treatment in landmark clinical trials,^1^, ^2^, ^3^, ^4^, ^5^, ^6^, ^7^ the median duration of ICI therapy in patients stopping ICIs because of durable efficacy was approximately 2 years in the present study. Contrary to expectations that most patients would receive single-agent pembrolizumab guided by favorable predictive factors such as high PD-L1 expression, only 5 of 23 patients (21.7%) were treated with this regimen. Furthermore, although irAEs have been associated with improved ICI efficacy,^21^ irAEs were relatively rare (17.4%) in these patients. This low incidence might partly reflect underreporting of irAEs in real-world clinical settings, where less rigorous monitoring and documentation compared to clinical trials could lead to an underestimation of their true frequency.

A previous retrospective study comparing continuous versus fixed 2-year duration ICI treatment in NSCLC reported that the continuous group tended to have more treatment-related AEs, with no significant difference in time to treatment failure >24 months.^19^ In the KEYNOTE-010 trial that showed improved OS with pembrolizumab compared with docetaxel in patients with previously treated NSCLC, approximately half of patients who had disease progression after stopping pembrolizumab after 2 years of treatment could be rechallenged with pembrolizumab. Importantly, high rates of objective response (52.3%) and disease control (81.0%) were achieved in the second-course pembrolizumab treatment.^29^ These results combined with the excellent survival outcomes observed following ICI discontinuation in the present study suggest that carefully assessed ICI cessation may be a viable strategy to reduce treatment burden and mitigate the risk of irAEs while preserving antitumor efficacy.

A meta-analysis reported that patients with NSCLC who discontinued ICIs for elective reasons had significantly longer post-discontinuation PFS compared with those who discontinued due to toxicity, whereas no significant difference was observed between these groups in patients with melanoma.^30^ Consistently, post-discontinuation survival outcomes were significantly worse in patients who discontinued ICIs because of irAEs compared with those who discontinued because of a response in the present study. Notably, when categorized by tumor response at the time of treatment discontinuation, patients in the irAE-CR/PR and irAE-SD-NE groups demonstrated comparable post-discontinuation OS outcomes, arguing the significance of response status in patients who stop treatment because of irAEs. Nevertheless, patients in the irAE-CR/PR group with ICI therapy duration of ≥12 months had improved post-discontinuation survival outcomes. This result highlights the association between duration of ICI exposure before discontinuation and post-discontinuation survival.

Our data are representative of a real-world clinical experience in a relatively large patient cohort. However, this study has some limitations. First, the number of patients in each category of ICI discontinuation was relatively small, particularly for those who discontinued ICIs because of a response. Consequently, we were unable to determine the optimal duration of ICI treatment for patients exhibiting durable responses. Although pivotal clinical trials have adopted upper limits of 2 years or 35 cycles administered every 3 weeks, treatment duration should vary among patients; this requires further investigation. Second, the data in this study were derived from four prospective cohort studies, resulting in heterogeneity in patient characteristics, including treatment types and lines of therapy. This variability would inherently introduce potential biases into the survival analysis. Third, although cohorts A–C enrolled patients with an ECOG PS of 0–2 and cohort D enrolled patients with any PS, the majority of patients in the entire cohort (92.4%) had an ECOG PS of 0 or 1. Therefore, it remains uncertain whether the results of this study could be directly extrapolated to real-world clinical settings. An additional limitation is the unavailability of key clinical and laboratory variables, such as PS, tumor burden, and LIPI, at the time of ICI discontinuation. The absence of these variables precluded the ability to perform a comprehensive multivariable analysis for post-ICI discontinuation survival, which may have provided deeper insights into prognostic factors influencing outcomes.

In summary, we analyzed real-world data to characterize the patterns of discontinuation among patients with advanced NSCLC receiving ICI therapy and evaluated survival outcomes based on discontinuation categories. To our knowledge, this is the first study to assess post-discontinuation survival outcomes in patients who discontinued ICIs due to irAEs with a specific focus on tumor response at the time of discontinuation. Our findings suggest that, despite being rare, discontinuation of ICIs because of a durable tumor response is a feasible strategy. Furthermore, ICI therapy lasting ≥12 months was associated with improved survival outcomes in patients who discontinued treatment due to irAEs and exhibited tumor response at the time of discontinuation. Further studies are warranted to validate our results and provide more insights for optimizing ICI therapy management in patients with advanced NSCLC.

## Disclosure

Dr. Inoue Speakers' Bureau: Chugai Pharma, AstraZeneca (outside the submitted work). Dr. Asada Speakers' Bureau: Chugai Pharma, AstraZeneca (outside the submitted work). The remaining authors declare no conflict of interest.
